# Ten-Year Probabilities of Death Due to Cancer and Cardiovascular Disease among Breast Cancer Patients Diagnosed in North-Eastern Spain

**DOI:** 10.3390/ijerph20010405

**Published:** 2022-12-27

**Authors:** Ramon Clèries, Alberto Ameijide, Maria Buxó, Mireia Vilardell, José Miguel Martínez, Rebeca Font, Rafael Marcos-Gragera, Montse Puigdemont, Gemma Viñas, Marià Carulla, Josep Alfons Espinàs, Jaume Galceran, Ángel Izquierdo, Josep Maria Borràs

**Affiliations:** 1 Pla Director d’Oncología, Av Gran Vía 199-203, 08908 L’Hospitalet de Llobregat, Barcelona, Spain; 2 Bellvitge Biomedical Research Institute, IDIBELL, Av. Gran Via de l’Hospitalet, 199-203-1a planta, 08908 L’Hospitalet de Llobregat, Barcelona, Spain; 3 Clinical Sciences Department, Universitat de Barcelona, 08907 Barcelona, Spain; 4 Tarragona Cancer Registry, Epidemiology and Cancer Prevention Service, Hospital Universitari Sant Joan de Reus, IISPV, 43204 Reus, Spain; 5 Girona Biomedical Research Institute, IDIBGI, C/Dr. Castany s/n, Edifici M2, Parc Hospitalari Martí i Julià, 17190 Salt, Spain; 6 Independent Researcher, 08700 Barcelona, Spain; 7 Statistics and Operational Research Department, Universitat Politècnica de Catalunya, EDIFICI H, Diagonal 647, 08028 Barcelona, Spain; 8 Public Health Research Group, University of Alicante, 03690 Alicante, Spain; 9 Girona Cancer Registry, Epidemiology Unit, Pla Director d’Oncologia, Institut Català d’Oncología, Group for Descriptive Epidemiology, Genetics and Cancer Prevention, Girona-IDIBGI, 17005 Girona, Spain; 10 Medical School, Universitat de Girona (UdG), 17071 Girona, Spain; 11 Epidemiology and Public Health Research Network Centre (CIBERESP), 28029 Madrid, Spain; 12 Medical Oncology Service, Catalan Institute of Oncology, Hospital Universitari de Girona “Doctor Josep Trueta”, 17005 Girona, Spain

**Keywords:** breast cancer, probability of death, cardiovascular disease, second tumours, causes of death, stage, hormone receptor status

## Abstract

Mortality from cardiovascular disease (CVD), second tumours, and other causes is of clinical interest in the long-term follow-up of breast cancer (BC) patients. Using a cohort of BC patients (N = 6758) from the cancer registries of Girona and Tarragona (north-eastern Spain), we studied the 10-year probabilities of death due to BC, other cancers, and CVD according to stage at diagnosis and hormone receptor (HR) status. Among the non-BC causes of death (N = 720), CVD (N = 218) surpassed other cancers (N = 196). The BC cohort presented a significantly higher risk of death due to endometrial and ovarian cancers than the general population. In Stage I, HR− patients showed a 1.72-fold higher probability of all-cause death and a 6.11-fold higher probability of breast cancer death than HR+ patients. In Stages II–III, the probability of CVD death (range 3.11% to 3.86%) surpassed that of other cancers (range 0.54% to 3.11%). In Stage IV patients, the probability of death from any cancer drove the mortality risk. Promoting screening and preventive measures in BC patients are warranted, since long-term control should encompass early detection of second neoplasms, ruling out the possibility of late recurrence. In patients diagnosed in Stages II–III at an older age, surveillance for preventing late cardiotoxicity is crucial.

## 1. Introduction

Breast cancer (BC) is a leading cause of cancer incidence and the second cause of cancer-related mortality among Spanish women [1]. Early diagnosis and advances in chemotherapy, hormonal treatment, and radiotherapy (RT) have led to improvements in five-year BC survival [2,3,4,5,6,7]. However, a small proportion of the women who survive BC beyond 5 years from diagnosis present a high probability of death from this cause within 10 years [8]. Moreover, evidence suggests that RT and chemotherapy are associated with an increased risk of cardiovascular disease (CVD) and second primary tumours [9,10,11,12,13].

Tumour stage at diagnosis is a major prognostic factor for the risk of death in BC patients [8], and it can also be useful for predicting cause-specific survival [12]. Stage data, however, usually cannot be used when comparing BC mortality between populations since not all population-based cancer registries collect it [3]. In the literature, BC is commonly divided into luminal A, luminal B, HER2 (human epidermal growth factor receptor 2)-enriched, and triple negative tumours [6,7,14,15]. Given that few population-based cancer registries worldwide routinely collect data on Ki-67, epidermal growth factor receptor, and cytokeratin testing [6,7,12,14,15], the BC subtypes are generally categorized as hormone receptor positive (HR+), based on expression of oestrogen and/or progesterone receptor; HER2-positive (HER2+), regardless of hormone receptor status; and triple-negative, lacking expression of HER2 and oestrogen or progesterone receptors [6,7]. Those BC subtypes each have distinct biological and clinical characteristics, including differences in risk factors, disease management, recurrence rates, and survival outcomes [6,7]. A personalized treatment approach is required based on the patient’s specific BC subtype [4,6,7,9]. The HR+ BC patients receive endocrine therapies based on tamoxifen and/or aromatase inhibitors, although a small proportion also receive chemotherapy [6,7]. Treatment for HER2+ BCs includes chemotherapy plus HER2-targeted antibodies or small-molecule inhibitor therapy, whereas triple-negative tumours receive chemotherapy alone [6,7]. In this line, in a previous study, we found no excess mortality in HR+ patients diagnosed beyond 49 years of age at Stage I or beyond 59 years at Stage II if they followed the prescribed endocrine treatment regimen [15]. As survival of BC improves, there is an increased interest in the long-term outcomes in these patients, including their cause-specific probabilities of death [15,16].

During the first 10 years of follow-up, BC remains the most common cause of death following a diagnosis of this cancer, followed by heart and cerebrovascular diseases [9,10,11,12,13,14,15,16]. Studies about mortality in BC cohorts with follow-up longer than 10 years have shown that the largest proportion of deaths may occur within 1 to 10 years after diagnosis, and the number of deaths decrease as more years pass after cancer diagnosis [9,10,11,12,13]. These studies have shown that cardiovascular, pulmonary, and gastrointestinal diseases; second tumours in the ovary and endometrium; and metastases to the liver and lung are the most common causes of death in BC patients [9,10,11,12,13]. Among the causes of non-BC death, evidence suggests that with longer follow-up, CVD could compete with cancer as the leading cause of death due to potential comorbid conditions in BC patients [13,17]. In a previous study, we analysed the causes of death in BC patients diagnosed from 1985 to 2004 using data from two historical population-based cancer registries in Girona and Tarragona (Spain) [12]. In that study, probabilities of death were calculated according to age, since those results could not be stratified by molecular subtype or stage, since only data on stage at diagnosis were available, and even then, for just half the cohort [12,16]. In the current study, we aim to estimate the probabilities of death due to cancer and CVD up to 10 years after BC diagnosis, stratifying results by age, stage, and hormone receptor status.

## 2. Materials and Methods

### 2.1. Data

We designed a population-based cohort study by using BC data obtained from population-based cancer registries in Girona and Tarragona (Catalonia, Spain), which covered an annual population of 771,294 women/year during the study period. All women aged 15–84 years and diagnosed with invasive primary BC from 2000 to 2009 in these regions were included. Cancer registry data included 6758 women diagnosed with invasive primary BC according to the International Classification of Diseases, 10th edition (ICD-10, code C50) from 2000 to 2009. Patients’ vital status was assessed up to 31 December 2019 through passive follow-up (record linkage between the cancer registries, the Mortality Registry of Catalonia, and the National Index of Deaths of the Spanish Ministry of Health) and active follow-up via hospitals. The person-years observed were defined as the period between BC diagnosis and death (if this occurred during the first 10 years of follow-up). In this line, if the patient was alive or died beyond 10 years after BC diagnosis, that patient contributed a maximum of 10 person-years. Moreover, if the patient died beyond 10 years after her BC diagnosis, the cause of death of that patient was not considered in the statistical analyses. The primary cause of death was retrieved from these sources and subsequently assessed using the ICD-10. To determine the exact cause of death in patients with cancer of unknown primary (C76–C80, C97), we reviewed the files of the cancer registries and the patient’s medical records.

Medical records were reviewed to extract data on TNM stage at diagnosis, according to the American Joint Committee on Cancer Staging Mannual, 7th edition [18]. HR and HER2 overexpression were recorded from pathology and clinical reports. Other variables collected were: age (24, 27, …, 83, 84), stage at diagnosis (5 categories: I/II/III/IV and Missing), follow-up years (1, …, 10), vital status (died vs survived), and cause of death, if applicable. Molecular subtype based on HR status was available only from the cohort diagnosed from 2005 to 2009, not in patients diagnosed earlier [16].

### 2.2. Statistical Methods

First, we assessed the cause-specific risk of death between the cohort and the general population. We also compared the risk of death between two diagnostic periods (2000–2004 versus 2005–2009) in order to assess whether the impact of CVD on the risk of death was different between periods. Second, we provided the 10-year probabilities of death due to causes in four broad categories: (i) BC, (ii) cancers other than breast cancer, (iii) CVD, and (iv) other causes. This statistical analysis is presented in two parts. The first presents results according to age and stage at diagnosis—information commonly available in cancer registry data. This analysis was performed using the whole cohort, since 92% of the patients had data on the stage of BC at diagnosis. The second part of the analysis assesses how adding data on HR status impacts the estimates of the aforementioned probabilities. Unfortunately, HR information was only available for the 2005–2009 cohort.

#### 2.2.1. Risk of Death Due to Causes Other Than BC Compared to the General Population

The relative risk of death due to causes other than BC in the cohort versus the general population was evaluated at 10 years’ follow-up by using the standardized mortality ratio (SMR), calculated as the ratio between the observed (O) number of deaths from a certain cause at 10 years from diagnosis of BC and the expected (E) deaths at that time if the patients in the cohort experienced the same mortality rates from that cause as the reference population [19]. Expected deaths were then calculated by applying the age and year-specific person-years at risk in the cohort to the corresponding cause-specific mortality rates of Catalonia, the reference population comprising the provinces of Girona and Tarragona. The patients not found to be dead beyond 10 years after their BC diagnosis were censored, each of these contributing 10 person-years.

Under the Bayesian framework, the posterior distribution of the SMR was estimated by assuming that O follows a Poisson distribution. Let *O_c_* be the observed number of deaths in the cohort for the *c*-th cause of death, and *E_c_* the corresponding expected number of deaths in the cohort. The Bayesian model for estimating a posterior distribution for the $SMR_{c}=\frac{Oc}{Ec}$ was defined as a log-linear hierarchical model as follows:

(1) $$O_{c}\sim Poisson\left(\mu_{C}\right)$$

(2) $$log\left(\mu_{C}\right)=log\left(E_{C}\right)+\theta_{c}$$

(3) $$\theta_{c}\sim N\left(0,\tau_{\theta }\right)$$

(4) $$\sigma_{\theta }\sim Uniform\left(1,5\right)$$

(5) $$\tau_{\theta }=\frac{1}{{\left(\sigma_{\theta }\right)}^{2}},$$

where $\mu_{C}$ is the theoretical mean of the observed number of deaths, and $\theta_{c}$ is a parameter representing the log-SMR for a Normal prior distribution with mean 0 and precision $\tau_{\theta }$ (inverse of the variance). Here, we imposed a uniform prior distribution over $\sigma_{\theta }$, with the standard deviation of $\theta_{c}$. This prior, using a lower bound of 1, has previously been proposed for dealing with a small number of cases and zero counts in the process of deriving a posterior distribution of the SMR [20]. The Bayesian model was implemented using WinBUGS [21] (see the programme code in supplementary material file), which was run within R (http://www.R-project.org, accessed on 25 May 2022) through the R2WinBUGS library [22]. The use of the Bayesian framework allowed us to provide three basic indicators for distinguishing the causes of death with significant impact: (i) the median SMR (SMR_Me_), (ii) the 95% credible interval (CrI) for the SMR, and (iii) the posterior probability that SMR > 1, P_SMR_. In the Bayesian framework, it is considered that a cause of death has a significant risk when that probability surpasses a certain threshold [23], in this case P_SMR_ > 0.95.

Moreover, we compared the SMRs between two cohorts, defined according to diagnostic period (2000–2004 versus 2005–2009), in order to assess cause-specific differences in the risk of death between periods. For this purpose, we calculated the ratio of the SMRs between diagnostic periods, Ratio = SMR_2_/SMR_1_, where SMR_1_ refers to the SMR calculated for the diagnostic period 2000–2004, and SMR_2_ to that calculated for 2005–2009. We also calculated the posterior probability that Ratio > 1, that is, P_Ratio_. The distribution of the ratio of SMRs was calculated using the Bayesian framework by resampling on the posterior distribution of SMR_2_ and SMR_1_.

The SMRs were evaluated for all-cause mortality, excluding BC, and for grouped causes of death according to ICD-10 disease groups: (i) tumours (C00–D49), (ii) diseases of the blood (D50–D89), (iii) endocrine organs (E00–E89), (iv) nervous system (G00–G99), (v) cardiovascular disease/circulatory system (CVD: I00–I99), (vi) respiratory system (J00–J99), (vii) digestive system (K00-K95), (viii) musculoskeletal system (M00–M99), (ix) genitourinary system (N00–N99), and (x) malformations and other unclassified findings (Q00–Q99). We also compared the SMRs between diagnostic periods as the ratio between SMRs. We calculated the probability that this ratio > 1 between periods.

#### 2.2.2. 10-Year Cause-Specific Probabilities of Death in the Cohort: Competing Risks Analysis

In survival analysis, competing risks are defined as events that may alter the probability of a primary outcome occurring during the follow-up of the cohort of patients [24]. If the primary event of interest is death due to cancer, then death due to CVD is a competing risk, since people who die of CVD are no longer at risk of death due to cancer. These events are considered informative since the risk of the cause-specific mortality changes after the competing risk occurs. In Kaplan–Meier analysis, censoring of competing risks artificially inflates the risk estimates, thus leading to the concept of immortal time (competing-risk) bias [24,25,26,27,28,29]. There are two options to overcome this issue: (i) the Fine–Gray subdistribution hazard model and (ii) cause-specific hazard models (CSH), estimated through Cox modelling, for each of the different event types [25,26,27,28,29]. The Fine and Gray model presents the known limitation that the sum of the combined probability estimates of the different competing events may surpass 1, which is not possible, but this issue is solved when using the CSH models [25,26,27,28,29]. CSH models combine the resulting cause-specific hazard models to estimate the absolute risk of each of the different event types over time.

We used the cumulative incidence/mortality competing risks (CIMCR) method [28] through CSH modelling, assuming that patients who experienced a competing cause of death were no longer at risk for the endpoint of interest [29]. This approach estimates the actual probabilities of reaching different endpoints (cumulative incidence/mortality) where, at each time point, the sum of all cumulative incidence/mortality will be equal to the total probability of reaching an endpoint before that time [27]. From this model, we estimated the cumulative probability of death considering four competing causes of death: BC, cancers other than BC, CVD, and other causes of death.

Let PBC(T) be the cumulative probability of dying from BC up to time T, with maximum T = 10 years, PCa(T) the cumulative probability of dying from cancers other than BC up to time T, PCVD(T) the cumulative probability of dying from CVD up to time T, and POC(T) the cumulative probability of dying from other causes up to time T. Let Total(T) be the cumulative probability of dying from any cause up to time T,

Total(T) = PBC(T) + PCa(T) + PCVD(T) + POC(T)(6)

Then, if we let S(T) be the probability of surviving beyond T, Total(T) = 1 − S(T), since S(T) + PBC(T) + PCa(T) + PCVD(T) + POC(T) = 1. These are the quantities estimated through CIMCR modelling of the causes of death. The Total(T) calculation is also referred to as the “absolute risk” calculation in the literature [24,25,26,27,28,29]. Analyses were performed with R software version 4.2.0 (www.cran-r.org, accessed on 25 May 2022) using the package “mstate” [29]. In this modelling, the age variable was categorized into three groups: ≤49, 50–68, and ≥69 years at BC diagnosis. For the last cohort, the age-adjusted hazard ratios obtained through the CSH modelling were used as estimates of relative risks (RR) when comparing the risk of death in HR− vs. HR+ patients across the stages of BC at diagnosis.

## 3. Results

### 3.1. Descriptive Analysis

Table 1 presents the clinical and pathological characteristics of the observed cohort in Girona and Tarragona, comparing diagnostic periods 2000–2004 versus 2005–2009. Up to 70.4% of the patients were HR+, whereas 4.8% of the patients had no available data on HR status, precluding their classification into a molecular subtype. The mean age at diagnosis was 59.1 years overall. Table 1 also shows a comparison of cohort characteristics according to a chi-square test. When differences were detected, a pairwise proportional comparison between group levels with corrections for multiple testing was calculated. Analyses showed that there was a larger proportion of patients diagnosed at Stage II in 2000–2004 (39.0%) than in 2005–2009 (34.5%) (*p* < 0.001). Moreover, a lower proportion of patients diagnosed in 2005–2009 died from BC (21.5%) compared to those diagnosed in 2000–2004 (15.9%) (*p* < 0.001). Likewise, the proportion of patients who died from any cause was higher in the 2000–2004 cohort (40.7%) compared to the 2005–2009 cohort (27.1%) (*p* < 0.001). Finally, most of the BC patients diagnosed in 2005–2009 were HR+ (70.4%), and only 4.8% could not be classified according to molecular subtype. Among the non-BC causes of death (N = 720), the number of deaths due to CVD (N = 218) surpassed those due to other cancers (N = 196).

Table 2 presents the SMRs for the causes of death other than BC at 10 years after BC diagnosis. Significantly higher mortality was detected for two groups of causes: (i) cancers other than BC, although this risk of death slightly decreased between diagnostic periods (SMR 2000–2004: 1.34; SMR 2005–2009: 1.24), and (ii) cardiovascular mortality, which was not significant in 2000–2004 but was in 2005–2009 (SMR = 1.32), showing a significant increase between diagnostic periods (P_Ratio_ = 0.97 > 0.95). With respect to cancer mortality (see Supplementary Table S1), both cohorts carried a high risk of death (P_SMR_ > 0.95) due to endometrial cancer (SMR 2000–2004: 3.23; SMR 2005–2009: 2.52), and the 2005–2009 cohort due to ovarian cancer (SMR: 1.93). The risk of liver cancer mortality was slightly higher in the later cohort (SMR_1_: 1.38; SMR_2_: 1.51; Ratio SMRs: 1.09), but this difference was not statistically significant (P_Ratio_ = 0.93). With respect to CVD mortality, the risk of death was significantly higher in the 2005–2009 cohort (Supplementary Table S2) due to the high risk of death from heart failure (N = 23; SMR 2005–2009: 1.94) and heart disease (N = 28; SMR 2005–2009: 1.72). Cerebrovascular disease-related deaths (N = 15) were among the most important causes of non-cancer deaths, but its SMR was not statistically significant.

### 3.2. 10-Year Cause-Specific Probabilities of Death in the Cohort

#### 3.2.1. Probabilities of Death According to Age and Stage at Diagnosis

**Figure 1:**
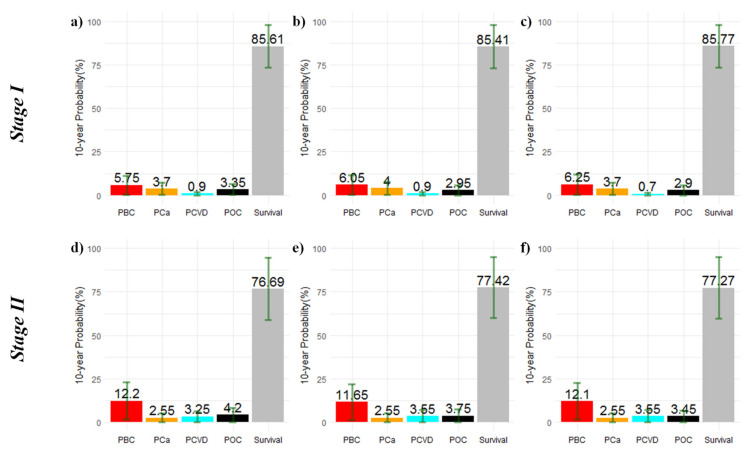
Cause-specific 10-year cumulative probabilities of death and probability of survival beyond 10 years after breast cancer diagnosis in Stages I–II. Panels show the results stratified by age group for the whole cohort: ≤49 (**a**,**d**), 50–68 (**b**,**e**), and ≥69 years (**c**,**f**). **Note:** vertical lines in dark green represent the standard deviation of the probability estimates.

**Figure 2:**
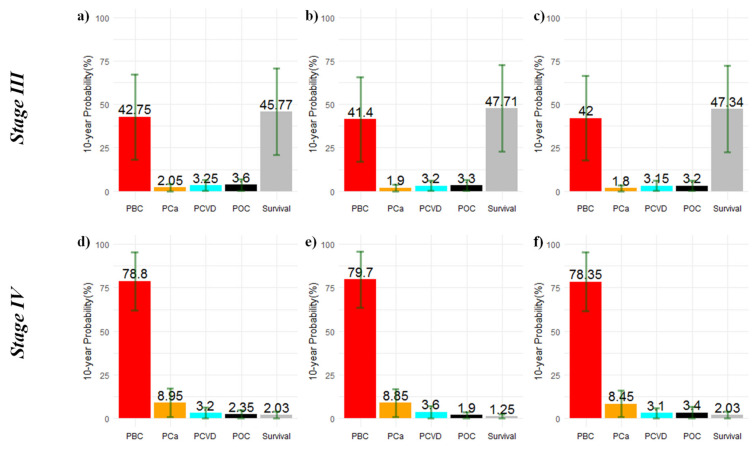
Cause-specific 10-year cumulative probabilities of death and probability of survival beyond 10 years after breast cancer diagnosis in Stages III–IV. Panels show the results stratified by age group for the whole cohort: ≤49 (**a**,**d**), 50–68 (**b**,**e**), and ≥69 years (**c**,**f**). **Note:** vertical lines in dark green represent the standard deviation of the probability estimates.

Figure 1 and Figure 2 depict the distribution of the 10-year PBC, PCa, PCVD, and POC as well as the probability of surviving beyond 10 years according to stage and age at BC diagnosis. BC was the main cause of death; however, in Stage I, the sum of probabilities of death due to non-BC surpassed the PBC (Figure 1a–c). In Stages II (Figure 1d–f) and III (Figure 2a–c), PCVD surpassed PCa, whereas in Stage IV (Figure 2d–f), the sum of the probabilities of death clearly surpassed the probability of survival beyond 10 years after BC diagnosis.

#### 3.2.2. Probabilities of Death According to Stage at Diagnosis when Hormone Receptor Data Is Available

Making use of the 2005–2009 BC cohort data, Table 3 presents the RR of death in HR− versus HR+ patients across stages of BC at diagnosis and adjusted for age as a continuous variable. The all-cause mortality modelling shows that RR differences are clearly marked between stages, ranging from 1.08-fold higher in HR− patients diagnosed in Stage IV to 1.72-fold higher in Stage I. When inspecting cause-specific mortality, BC is the main driver of these RRs. Cause-specific modelling in this table is presented in order to illustrate (i) the impact of BC mortality and (ii) the impact of all causes of mortality excluding BC. For cause-specific modelling, we aggregated the causes of death other than BC and compared these risks of death with those obtained with BC as cause of death. Differences between RRs are also marked, where the largest RR is observed in Stage I (6.11-fold higher risk in HR− patients), followed by Stages II–III (1.62 and 1.68-fold higher) and Stage IV (1.29-fold higher). When inspecting non-BC mortality, all 95% confidence intervals of the RRs included the value of 1, indicating non-significant RRs. The age-adjusted cumulative probability of incidence and survival curves, stratified by hormone receptor status and stage at diagnosis, are depicted in Supplementary Material Figure S1.

The 10-year cause-specific probabilities of death according to age group, stage, and hormone receptor status are presented in Table 4 (Supplementary Material Tables S3.1–S3.4 include the 95% confidence intervals for these probabilities, showing the large variability in the estimates). Our modelling shows that the largest difference in these probabilities is in Stage I patients, where the total probability of death in HR− patients is clearly higher than in HR+ patients (ratio 1.66 to 1.80, depending on the age group). This result is mainly due to the differences in PBC mortality between HR− and HR+ patients across age groups (ratios for ages ≤49: 5.36; ages 50–68: 6.09; ages ≥69 years: 6.33). The PBC is surpassed by the sum of the probabilities of death due to any other cause in HR+ patients diagnosed in Stages I–II. In Stages II–III, PCVD (range 3.11% to 3.86%) surpasses PCa (range 0.54% to 3.11%). On the contrary, PCa is the second cause of death in Stage IV patients, surpassing PCVD.

## 4. Discussion

Knowledge on non-BC causes of death might help to understand the impact of other diseases and second cancers on long-term survival in patients diagnosed with BC, taking into account factors other than treatment (only available for a few cases in our current dataset). In our cohort, of the total 720 non-BC causes of death, 218 (30.3%) were due to CVD, and 196 due to other cancers (27.2%). In patients diagnosed in Stages II–III, the 10-year PCVD (range 3.11% to 3.86%), surpassed the 10-year PCa (range 0.54% to 3.11%). In Stage I patients, the 10-year probability of death was at least 1.66-fold higher in HR− compared to HR+ patients.

Previous studies evaluating non-BC deaths in BC patients have shown that other cancers, mainly metastases to the liver, lung, and brain, as well as CVD, pulmonary, and gastrointestinal diseases, contributed to the increased risk of death [9,10,11,12,13,17,30,31]. Below, we discuss our results around three categories of causes of death: (i) cancer, (ii) CVD, and (iii) other causes, focusing on the role of CVD as a competing risk of death to cancer. Of note, CVD was the leading cause of death in the general population of Girona and Tarragona from 2010 to 2019 (mean 1735 deaths/year, or 27.3% of all deaths during that study period), followed by any form of tumour (N = 1410, 22.2%) and diseases of the respiratory system (N = 606, 9.6%) (See the Supplementary Table S4, adapted from Catalan Institute of Statistics website, IDESCAT; www.idescat.cat, accessed on 1 May 2022). This ranking of the three main causes of death in the general population is similar to the one that can be derived from Table 1.

### 4.1. Cancer Causes

Mortality risk was higher in the BC cohort than in the general population for endometrial and ovarian cancer, a result consistent with our previous studies of the risk of second tumours after BC diagnosis [32]. The BC patients in our cohort who died from endometrial cancer were diagnosed with BC, on average, at 70 years of age (see Supplementary Material Figure S2), older than the BC patients in our cohort who died from BC (close to 60 years of age). Endogenous and exogenous hormones could play a role in this outcome [33], as could potential surveillance bias after the diagnosis of BC or tamoxifen treatment, which is related to the development of endometrial hyperplasia and cancer [32,33,34,35,36,37]. Regarding oestrogen receptor or progesterone status [38], common aetiological factors among BC subtypes might also increase the risk of endometrial cancer [39,40,41,42]. In this line, some studies have discussed the link between that cancer and shared reproductive factors, such as nulliparity [39] and other risk factors [43], in addition to late age at BC diagnosis [39,44]. On the other hand, several studies of second tumours after BC treatment have also reported on the risk of ovarian cancer mortality [39]. Increased mortality due to ovarian cancer can be explained by shared inherited susceptibility genes such as BRCA1 or BRCA2 [45], especially in young women [40,45,46,47].

In our cohort, we did not detect a significant risk of death from lung cancer. However, evidence suggests an association between lung cancer as a second tumour after BC and side effects of RT [48], where the risk factors are age at BC diagnosis, smoking, chemotherapy, and RT [49]. In autopsy studies on metastatic patterns of BC, 57% to 77% of BC patients, mostly triple-negative or HER2−, have lung metastases [50]. The proportion of women with locoregional BC treated with RT has increased, while the volume of non-breast irradiated tissues has decreased [51]. However, substantial radiation exposure in organs other than the treated BC—a small part of the heart, lung, and bone marrow, which often receive doses as high as 50 Gy—cannot be avoided and could potentially induce second primary cancer or heart disease [51,52]. Moreover, for lung cancer, smoking intensity is an important risk factor, and smoking cessation can reduce in radiation-induced rates of lung cancer after BC [51].

Patients diagnosed in the second period had a higher risk of liver cancer mortality compared to the first, but this difference was not statistically significant. Some studies have shown that the risk of liver metastasis is second only to bone and lung metastasis [53]. Young age at diagnosis is a risk factor for liver metastases [54], and in our study, two out of every five women who died of liver cancer in 2005–2009 had been diagnosed with BC before the age of 52 years. Hepatic lesions in BC patients are frequently considered a metastatic site [55], so second primary liver cancers in BC patients must be considered during the clinical follow-up.

We did not detect a higher risk of death due to pancreatic cancer, although this tumour ranked as the fourth most common cause of cancer death in the cohort. This is a rare outcome [56], since the link between BC and pancreatic cancer has been reported for advanced-stage BC [57] and related through rare BRCA1 and BRCA2 mutations [58,59]. Unfortunately, BRCA data were not available, so further research is still necessary.

The cohort did not show a significantly higher risk of death from colon cancer, the second cause of cancer death, compared to the general population, but the increasing risks of developing this cancer in Spanish women aged 65 and older [52] (second tumour in the ranking of cancer incidence in Catalonia in women aged 74 and older), together with the good survival prospects of BC [15], are factors suggestive of an elevated future risk of colorectal incidence in our cohort. In contrast, the downward trend for risk of death due to leukaemia and haematological malignancies detected in our previous study [12] is consistent with our current results, since these tumours showed a non-significant risk relative to the general population.

### 4.2. Cardiovascular Mortality

CVD emerges as the second competing risk of death among BC survivors after BC. The risk of death from heart diseases in BC patients has been clearly established in a large cohort study carried out in the USA [17]. In the 2005–2009 cohort, deaths from acute myocardial infarction (n = 13), other ischaemic diseases of the heart (n = 12), heart failure (n = 23), and other heart diseases (n = 28) were the largest contributing causes to CVD mortality. Our results show that the risk of death from CVD—mainly heart disease—was related to age, since 90% of our cohort who died due to CVD were over 75 years of age when they were diagnosed with BC and had a longer follow-up than the patients who died from the other significant causes of death (see Supplementary Material Figure S2), which is consistent with other studies carried out in the USA [17]. In another recent study in the USA, the time trend of CVD mortality risk increased in regional-advanced BCs treated with chemotherapy and/or RT [60]. BC patients receiving RT are at increased risk of heart disease [17], myocardial infarction [31], coronary atherosclerosis, cardiomyopathy, and valvular dysfunction [61]. A large cohort study comparing the risks of cardiac mortality in patients diagnosed before 2002 showed a significant impact in young BC patients receiving RT, and this risk increased with chemotherapy [62]. On the other hand, aromatase inhibitors may be linked to endothelial dysfunction, which increases the incidence of CVD [63,64], while anthracyclines and HER2-targeted antibodies can cause cardiotoxic effects [65]. These treatments might increase the risk of adverse events such as heart failure and cardiomyopathy [66,67] in regional stage patients treated with chemotherapy alone, highlighting the need for additional studies with detailed treatment data and cardiovascular management throughout cancer survivorship. Some cardiovascular and cerebrovascular deaths may be related to the effects of adjuvant anti-oestrogen therapy prescribed after BC diagnosis [13,68]. While triple-negative BC treatment combining RT with breast-conserving surgery, such as a lumpectomy, can be very effective [69], side effects such as atherosclerosis [61] should be monitored during patient follow-up.

### 4.3. Other Causes of Death

Certain studies have detected an increased risk of endocrine, nutritional, and metabolic disorders, including abdominal adiposity, insulin resistance, hyperglycaemia, hypertension, and dyslipidaemia [70,71]. In our cohort, all deaths from these diseases were diagnosed in BC patients over 65 years of age, so the combination of menopause and metabolic disease must be considered a prognostic factor for BC patients. Moreover, a recent meta-analysis reported that women with diabetes are at higher risk for BC-specific and all-cause mortality after BC diagnosis [72]. The potential impact of HR and menopausal status on endocrine, nutritional, and metabolic diseases and BC prognosis requires further research [70]. On the other hand, unlike in our previous study, we did not observe an increased risk of death from genitourinary diseases [12]. Our results in the current study are in the line with recent studies [73].

### 4.4. Cardiovascular Disease as the Competing Risk of Death to Cancer

Our results show that the stage of BC is the factor most clearly related to survival, but a competing risk analysis presenting the probabilities of death by large groups of causes is crucial for assessing survival in BC patients [74]. In Stage I, the probability of death from any cause is 1.72-fold higher, and from BC 6.11-fold higher, in HR− compared to HR+ patients, and this result is consistent across age groups. In HR+ patients diagnosed in Stages I–II, we found that the 10-year probability of death due to BC was surpassed by the sum of the probabilities of the remaining competing causes of death. A similar result was detected in another study carried out in Australia [75], suggesting that the effect of specific comorbidities and treatment may have an effect on this difference between probabilities. In a previous study, we showed the positive impact of endocrine therapies in the survival prospects of HR+ BC patients diagnosed in early stages of BC: better survival than HR− patients, and no excess mortality up to 10 years after BC diagnosis [15]. However, that study estimated crude probabilities of death due to any other cause than BC and could not provide estimates of cause-specific probabilities. Here, by using the competing risk methodology, we found that mortality from CVD is the major competing risk to BC mortality in women diagnosed in Stages II–III: PCVD (range 3.11% to 3.86%) surpassed PCa (range 0.54% to 3.11%). This finding is consistent across age groups and HR status, adding evidence to the results of previous studies reporting that the risk of death from CVD might surpass the risk of death from any cancer other than BC in women diagnosed with early-stage BC at advanced ages [75,76,77]. In these studies, pre-existing comorbidities and CVD increased CVD mortality among BC patients, even though BC was the main cause of death overall [78,79]. In advanced stages of BC, the probability of death from any cancer drives the risk of death among BC patients [78]. Previous studies have noted that biological characteristics may vary in distinct metastatic patterns with a clear impact on BC survival, decreasing the risk of death from any cancer but clearly surpassing the risk of death from non-cancer causes [80,81,82].

### 4.5. Strengths and Limitations

The study’s two main strengths are (i) the population-based nature of the data, which minimizes the risk of bias, and (ii) the long-term follow-up, considering molecular subtype and stage at diagnosis, crucial predictors of BC survival. To our knowledge, this is the first study presenting cause-specific mortality risks in BC patients that accounts for these factors up to 10 years after BC diagnosis. Worth noting is that all patients had a potential follow-up of 10 years.

Information on endocrine treatment for HR+ BCs diagnosed in Stages I–III was not available for all these patients [14,15], constituting a limitation for interpreting the results of our study. However, the benefit of receiving endocrine treatment in HR+ patients at early stages can be partially assessed, since these patients have shown a lower probability of death compared to HR− patients. The non-availability of chemotherapy and radiotherapy data in our study is a limitation given its relation to all-cause death [60]. In this line, information on the treatment pathway, surgery information, or other strategies can also be used to assess changes in CVD risk or cancer prevalence. This information is of interest and must be collected in future mortality studies in BC patients. Finally, another limitation lies in the quality of the death certificates; additional classification errors may also stem from cause-of-death coding in the cancer registry [83,84,85]. In addition, our databases and study design precluded assessing risk of death by contralateral and bilateral BC.

We must note that our aim was to assess the 10-year risk of death in a cohort of BC patients, and the maximum date considered for follow-up was 31 December 2019. We chose this date since the impact of COVID-19 pandemic on Spanish mortality statistics must be specifically evaluated. A recent study has suggested that the analysis of mortality for cancer cohorts in Spain followed up beyond the year 2019 must be performed separately by time periods (time period 1: up to 2019/time period beyond 2019) [86].

## 5. Conclusions

CVD is the second competing risk of death among BC survivors after BC mortality, with an especially marked impact in patients diagnosed in Stages II–III. However, (non-BC) cancer mortality remains the second cause of death after BC in Stages I and IV. In this line, BC patients are at a significantly higher risk of death from endometrial and ovarian cancer compared to the general population. Differences in the risk of death according to HR status have been marked in patients in Stage I, where HR− patients presented 1.72-fold and 6.11-fold higher probabilities of all-cause and BC mortality, respectively, than HR+ patients.

These results show that BC patients diagnosed in Stages II–III at an older age may be at a high risk of dying from CVD, so surveillance and prevention of late cardiotoxicity are crucial for this population, and promoting adherence to screening and preventive measures is warranted.

## Figures and Tables

**Table 1:** Characteristics of patients diagnosed with breast cancer from 2000 to 2009 in Girona and Tarragona.

	Total cohort 2000–2009 (N = 6758)	Diagnosed 2000–2004 (N = 3296)	Diagnosed 2005–2009 (N = 3462)	*p* Value
**Registry**				
Girona	3213 (47.5%)	1549 (47.1%)	1664 (48.1%)	0.77
Tarragona	3545 (52.5%)	1747 (52.9%)	1798 (51.9%)	
**Age**				
Mean (SD)	59.1 (14.3)	59.7 (14.9)	58.6 (13.6)	0.35 (a)
**Age group**				
≤49	1880 (27.8%)	863 (26.2%)	1017 (29.4%)	0.18
50–68	3718 (55.0%)	1844 (55.9%)	1874 (54.1%)	
≥69	1163 (17.2%)	592 (17.9%)	571 (16.5%)	
**Stage**				
I	2257 (33.9%)	1084 (32.9%)	1173 (33.9%)	<0.01
II	2478 (36.7%)	1284 (39.0%)	1194 (34.5%)	
III	1080 (16.0%)	475 (14.4%)	605 (17.5%)	
IV	463 (6.8%)	208 (6.3%)	255 (7.4%)	
Missing	484 (7.1%)	249 (7.4%)	235 (6.7%)	
**Cause of death**				
Breast cancer	1296 (19.2%)	744 (21.5%)	552 (15.9%)	<0.01
Other cancer (a)	196 (2.9%)	95 (2.7%)	101 (3.0%)	
Cardiovascular disease	218 (3.2%)	112 (3.2%)	106 (3.2%)	
Other causes	306 (4.5%)	136 (3.9%)	170 (4.9%)	
Alive	4582 (67.8%)	2056 (59.3%)	2526 (72.9%)	
**Follow-up (years)**				
Mean (SD)	8.3 (2.9)	8.0 (3.1)	8.6 (2.7)	0.33 (a)
**Molecular subtype**				
HR+	2748 (40.6%)	*	2748 (70.4%)	(b)
HR−	547 (8.1%)	*	547 (15.8%)	
Unknown	3463 (51.2%)	*	167 (4.8%)	

(a): *p* value for *t*-test, correcting for heterogeneity of variance; *: data not available; (b): cannot be calculated.

**Table 2:** Comparison of the SMRs between the 2000–2004 cohort and the 2005–2009 cohort according to the grouped causes of death, excluding breast cancer deaths.

	2000–2004 Cohort				2005–2009 Cohort				Ratio	
Cause	O_1_	%	SMR_1_	P_SMR1_	O_2_	%	SMR_2_	P_SMR2_	SMR_2_/SMR_1_	P_Ratio_
All causes (except breast cancer)	337	100.0	1.16	0.98	365	100.0	1.14	0.97	0.98	0.14
Infectious diseases	7	2.1	1.22	0.66	7	1.9	1.07	0.53	0.88	0.35
Tumours (except breast)	95	28.2	1.34	0.98	101	27.7	1.24	0.97	0.92	0.22
Diseases of the blood/hematopoietic organs	1	0.3	0.34	0.05	1	0.3	0.51	0.14	1.50	0.75
Endocrine, nutritional-metabolic diseases	13	3.8	1.17	0.67	15	4.1	1.41	0.88	1.21	0.69
Mental and behavioural disorders	14	4.2	0.79	0.15	17	4.7	0.69	0.04	0.87	0.36
Diseases of the nervous system/organs of the senses	20	5.9	1.07	0.59	20	5.5	0.77	0.10	0.72	0.21
Cardiovascular disease/circulatory system	112	33.2	1.13	0.90	106	29.0	1.32	0.99	1.17	0.97
Diseases of the respiratory system	21	6.2	0.85	0.21	37	10.1	1.24	0.89	1.46	0.91
Diseases of the digestive system	20	5.9	1.37	0.91	17	4.7	0.74	0.08	0.54	0.12
Diseases of the skin and subcutaneous tissue	1	0.3	0.67	0.22	2	0.6	1.96	0.73	2.93	0.81
Musculoskeletal system and connective tissue	4	1.2	1.50	0.72	8	2.2	1.68	0.79	1.32	0.70
Diseases of the genitourinary system	11	3.3	1.38	0.82	18	4.9	1.20	0.75	0.87	0.37
Congenital malformations	4	1.2	5.72	0.99	0	0.0	0.00	0.36	0.00	0.14
Symptoms, signs, and abnormal findings	7	2.1	1.30	0.72	8	2.2	1.38	0.77	1.06	0.54
External causes of mortality	7	2.1	1.01	0.47	8	2.2	0.97	0.40	0.96	0.46

**SMR_1_:** standardized mortality ratio for patients diagnosed in 2000–2004; **P_SMR_:** probability that SMR > 1; **SMR_2_:** standardized mortality ratio for patients diagnosed during 2005–2009; **Ratio:** Ratio SMR_2_/SMR_1_; **P_Ratio_:** probability that Ratio > 1.

**Table 3:** Relative risks derived from the all-cause mortality and the cause-specific modelling analysis.

	Cox Modelling *	Cause-Specific Hazard (CSH) **	
	All-Cause	BC	Non-BC
	Mortality	Mortality	Mortality
	RR (HR− vs HR+)	RR (HR− vs HR+)	RR (HR− vs HR+)
Dataset	95% CI	95% CI	95% CI
Stage I	1.72 (1.08; 2.77)	6.11 (1.97; 9.59)	0.76 (0.35; 1.37)
Stage II	1.18 (1.09; 1.79)	1.62 (1.13; 3.63)	0.95 (0.78; 2.22)
Stage III	1.38 (1.11; 1.96)	1.68 (1.21; 3.85)	0.67 (0.26; 2.72)
Stage IV	1.08 (1.02; 1.35)	1.29 (1.08; 4.44)	0.75 (0.42; 1.47)

**CI:** confidence interval; **RR:** relative risk estimated through modelling; *: model adjusted for age group and HR status; **: The CSH model in this table was fitted using 2 competing causes of death, BC vs. non-BC (other cancer, CVD and OC). Model adjusted for age group and HR status.

**Table 4:** 10-year cause-specific probabilities of death according to age, stage, and hormone receptor status in the cohort of patients diagnosed in 2005–2009.

		Age at Diagnosis of BC														
		≤49 Years					50–68 Years					≥69 Years				
		PBC (%)	PCa (%)	PCVD (%)	POC (%)	Total (%)	PBC (%)	PCa (%)	PCVD (%)	POC (%)	Total (%)	PBC (%)	PCa (%)	PCVD (%)	POC (%)	Total (%)
**Stage I**	**HR+**	1.81	3.71	1.21	3.31	10.04	1.71	3.64	1.42	3.21	9.98	1.71	4.11	1.31	3.12	10.25
	**HR−**	9.71	3.72	0.61	3.40	17.44	10.42	4.42	0.41	2.71	17.96	10.82	3.33	0.12	2.71	16.98
**Ratio**	(**−/+**)	5.36	1.00	0.50	1.03	1.74	6.09	1.21	0.29	0.84	1.80	6.33	0.81	0.09	0.87	1.66
**Stage II**	**HR+**	9.31	2.51	3.23	5.14	20.19	9.01	2.04	3.01	5.01	19.07	9.61	2.41	3.41	4.42	19.85
	**HR−**	15.12	2.61	3.34	3.32	24.39	14.11	2.82	3.86	2.43	23.22	14.65	2.72	3.71	2.51	23.59
**Ratio**	(**−/+**)	1.62	1.04	1.03	0.65	1.21	1.53	1.21	1.17	0.48	1.16	1.52	1.13	1.09	0.57	1.19
**Stage III**	**HR+**	32.21	2.91	3.23	3.75	42.10	32.99	2.91	3.11	3.82	42.83	32.91	3.11	3.11	4.28	43.41
	**HR−**	53.34	1.23	3.31	3.52	61.40	49.94	0.94	3.31	2.84	57.03	52.15	0.54	3.25	2.77	58.71
**Ratio**	(**−/+**)	1.66	0.42	1.02	0.94	1.41	1.51	0.32	1.06	0.74	1.33	1.58	0.17	1.05	0.65	1.38
**Stage IV**	**HR+**	70.31	12.43	3.16	3.39	89.29	72.61	12.22	3.43	2.76	91.02	70.61	13.76	2.84	4.75	91.96
	**HR−**	87.35	5.57	3.33	1.44	97.69	86.82	5.56	3.85	1.15	97.38	87.14	5.21	3.43	2.12	97.90
**Ratio**	(**−/+**)	1.24	0.45	1.05	0.42	1.09	1.20	0.45	1.12	0.42	1.07	1.23	0.38	1.21	0.45	1.10

**HR+:** hormone receptor positive patients; **HR−:** hormone receptor negative patients, as HER2-enriched and Triple Negative BCs.

## Data Availability

The data that support the findings of this study are available from the corresponding author upon reasonable request.

## Supplementary Materials

The following supporting information can be downloaded at: https://www.mdpi.com/article/10.3390/ijerph20010405/s1, Table S1: the complete table of cause-specific mortality; Table S2: the standardized mortality ratios for cardiovascular mortality in the cohort; Table S3.1: ten year probabilities of death and their corresponding 95% confidence interval according to age and hormone receptor status among patients diagnosed in Stage I; Table S3.2: ten year probabilities of death and their corresponding 95% confidence interval according to age and hormone receptor status among patients diagnosed in Stage II; Table S3.3: ten year probabilities of death and their corresponding 95% confidence interval according to age and hormone receptor status among patients diagnosed in Stage III; Table S3.4: ten year probabilities of death and their corresponding 95% confidence interval according to age and hormone receptor status among patients diagnosed in Stage IV; Table S4, causes of death in Catalonia among women during the period 2010–2019; Figure S1: Age-adjusted cumulative cause-specific mortality and survival probabilities accross stages of breast cancer at diagnosis and stratifying according to hormone receptor status. Figure S2: Ellipses representing the 95% confidence interval for the age at diagnosis of BC versus the number of years to develop/die from a certain disease (follow-up) for the significant causes of death.
