# Supplementary material for: Ten-Year Probabilities of Death Due to Cancer and Cardiovascular Disease among Breast Cancer Patients Diagnosed in North-Eastern Spain
Source: Int J Environ Res Public Health. 2022 Dec 27;20(1):405. doi: 10.3390/ijerph20010405 (PMC9819018; doi:10.3390/ijerph20010405)
Supplement: Supplementary file 1 [file ijerph-20-00405-s001.zip › ijerph-2059209-supplementary.pdf]

Supplementary material for

Ten-Year Probabilities of Death Due to Cancer and Cardiovascular Disease among Breast Cancer Patients Diagnosed in North-Eastern Spain

Ramon Clèries [1,2,3,*], Alberto Ameijide [4], Maria Buxó [5], Mireia Vilardell [6], José Miguel Martínez [7,8], Rebeca Font [1,2], Rafael Marcos-Gragera [5,9,10,11], Montse Puigdemont [9], Gemma Viñas [12], Marià Carulla [4], Josep Alfons Espinàs [1,2], Jaume Galceran [4], Ángel Izquierdo [9,12] and Josep Maria Borràs [1,2,3]

[1] Pla Director d'Oncología, Av Gran Vía 199-203, 08908 L'Hospitalet de Llobregat, Barcelona, Spain
[2] Bellvitge Biomedical Research Institute, IDIBELL, Av. Gran Via de l'Hospitalet, 199-203-1a planta, 08908 L'Hospitalet de Llobregat, Barcelona, Spain
[3] Clinical Sciences Department, Universitat de Barcelona, 08907 Barcelona, Spain
[4] Tarragona Cancer Registry, Epidemiology and Cancer Prevention Service, Hospital Universitari Sant Joan de Reus, IISPV, 43204 Reus, Spain
[5] Girona Biomedical Research Institute, IDIBGI, C/Dr. Castany s/n, Edifici M2, Parc Hospitalari Martí i Julià, 17190 Salt, Spain
[6] Independent Researcher, 08700 Barcelona, Spain
[7] Statistics and Operational Research Department, Universitat Politècnica de Catalunya, EDIFICI H, Diagonal 647, 08028 Barcelona, Spain
[8] Public Health Research Group, University of Alicante, 03690 Alicante, Spain
[9] Girona Cancer Registry, Epidemiology Unit, Pla Director d'Oncologia, Institut Català d'Oncología, Group for Descriptive Epidemiology, Genetics and Cancer Prevention, Girona-IDIBGI, 17005 Girona, Spain
[10] Medical School, Universitat de Girona (UdG), 17071 Girona, Spain
[11] Epidemiology and Public Health Research Network Centre (CIBERESP), 28029 Madrid, Spain
[12] Medical Oncology Service, Catalan Institute of Oncology, Hospital Universitari de Girona "Doctor Josep Trueta", 17005 Girona, Spain
* Correspondence: r.cleries@iconcologia.net; Tel.: +34-93-260-74-17

WinBUGS Code for calculating SMR

```
Model

{

# Obs: refers to O as it appears in the paper

# Esp: refers to E as it appears in the paper

# Likelihood


    Obs  ~ dpois(mu)

    theta~dnorm(0,tau.theta)

    log(mu)<-log(Esp)+theta

    SMR <- exp(theta)

    pp.SMR<-step(SMR-1)

    sigma.theta~dunif(1,5)

    tau.a<-1/pow(sigma.theta,2)


}
```

Supplementary Table S1. Comparison of the standardized mortality ratios for cancer mortality in the cohort of patients diagnosed during 2000-2004 versus the cohort of patients diagnosed during 2005-2009.

	------Cohort 2000-2004----				------Cohort 2005-2009----				Ratio	
**Cause**	O_1	%	SMR_1	P_{SMR1}	O_2	%	SMR_2	P_{SMR2}	SMR_2 / SMR_1	P_{Ratio}
**Tumours (except breast)**	95	28.2	1.34	0.98	101	27.7	1.24	0.97	0.92	0.22
Lip, oral cavity & pharynx	2	0.6	2.33	0.79	1	0.3	1.42	0.49	0.61	0.29
Stomach	7	2.1	1.65	0.87	5	1.4	1.05	0.49	0.64	0.22
Colon	14	4.1	1.45	0.89	14	3.8	1.14	0.65	0.79	0.35
Rectum	3	0.9	1.02	0.43	2	0.6	0.96	0.38	0.94	0.45
Liver & biliary tract	5	1.5	1.38	0.71	5	1.4	1.51	0.77	1.09	0.93
Pancreas	7	2.1	1.41	0.78	10	2.7	1.38	0.81	0.98	0.48
Other digestive tumours	1	0.3	0.38	0.07	2	0.6	0.70	0.22	1.84	0.71
Larynx	1	0.3	3.45	0.75	1	0.3	5.02	0.82	1.46	0.87
Trachea, bronchus & lung	9	2.7	1.54	0.87	12	3.3	1.35	0.82	0.88	0.39
Bone & articular cartilages	1	0.3	5.53	0.84	1	0.3	3.63	0.76	0.66	0.11
Skin & soft tissues	1	0.3	0.70	0.23	1	0.3	0.94	0.34	1.34	0.81
Cervix uteri	1	0.3	0.49	0.13	1	0.3	0.74	0.25	1.51	0.83
Endometrial (Corpus uteri)	9	2.7	3.23	0.99	8	2.2	2.52	0.99	0.78	0.21
Ovary	6	1.8	1.50	0.79	8	2.2	1.93	0.96	1.29	0.69
Other genital female organs	0	0.0	0.00	0.37	1	0.3	0.94	0.34	-	0.44
Kidney, except renal pelvis	1	0.3	0.43	0.10	3	0.8	1.98	0.81	4.60	0.85
Urinary bladder	1	0.3	0.31	0.04	3	0.8	1.61	0.72	5.19	0.87
Urinary tract	0	0.0	0.00	0.27	1	0.3	1.59	0.53	-	0.57
Encephalon	2	0.6	0.89	0.34	5	1.4	1.82	0.86	2.04	0.84
Other neuro/endocrine	1	0.3	1.53	0.52	1	0.3	1.24	0.44	0.81	0.15
Haematol. (non-leukaemia)	5	1.5	1.05	0.49	7	1.9	1.39	0.77	1.32	0.69
Leaukemia	6	1.8	2.04	0.92	5	1.4	1.70	0.83	0.83	0.38
Other and unknown	12	3.6	1.50	0.89	3	0.8	0.90	0.34	0.60	0.17

SMR_1: standardized mortality ratio for patients diagnosed during 2000-2004; **P:** probability; **SMR_2:** standardized mortality ratio for patients diagnosed during 2005-2009;

Supplementary Table S2. Standardized mortality ratios for cardiovascular mortality in the cohort of patients diagnosed during 2000-2004 and 2005-2009.

	Cohort 2000-2004				Cohort 2005-2009			
	**(O_1)**	**(%)**	**SMR_1**	**P_{SMR1}**	**(O_2)**	**(%)**	**SMR_2**	**P_{SMR2}**
**Circulatory system diseases (O=106)**	112	33.2	1.13	0.90	106	29.0	1.32	0.99
Chronic rheumatic heart diseases	3	0.9	1.35	0.75	2	0.5	1.25	0.65
Hypertensive diseases	9	2.7	0.79	0.14	8	2.1	0.72	0.15
Acute myocardial infarction	17	5.0	1.46	0.81	13	3.4	1.36	0.84
Other ischemic diseases of the heart	17	5.0	1.39	0.74	12	3.1	0.99	0.49
Heart failure	18	5.3	1.65	0.91	23	6.0	1.94	0.99
Other heart diseases	23	6.8	1.24	0.88	28	7.3	1.72	0.99
Cerebrovascular diseases	16	4.7	0.61	0.25	15	3.9	0.59	0.06
Atherosclerosis	4	1.2	1.67	0.78	2	0.5	1.60	0.71
Other diseases of blood vessels	5	1.5	0.99	0.48	3	0.8	0.84	0.38

SMR_1: standardized mortality ratio for patients diagnosed during 2000-2004; **P_{SMR1}:** probability SMR_1

SMR_2: standardized mortality ratio for patients diagnosed during 2005-2009; **P_{SMR2}:** probability SMR_2

Supplementary Table S3.1. Ten year probabilities of death and their corresponding 95% confidence interval according to age and hormone receptor status among patients diagnosed in Stage I.

Age<br><br>Years		PBC<br><br>(%, 95%CI)	PCa<br><br>(%, 95%CI)	PCVD<br><br>(%, 95%CI)	POC<br><br>(%, 95%CI)	Total<br><br>(%, 95%CI)
**≤49**	HR+	1.81 (0.35; 3.31)	3.71 (1.62;5.77)	1.21 (0.24;2.29)	3.31 (1.24;5.17)	10.04 (6.91;13.11)
	HR-	9.71 (6.76;12.68)	3.72 (1.27;5.39)	0.61 (0.05;1.31)	3.40 (1.18;5.63)	17.44 (14.01;20.82)
	Ratio (+/-)	5.36 (2.29; 7.79)	1.00 (0.01;1.95)	0.50 (0.00;1.03)	1.03 (0.25;1.97)	1.74 (1.25;2.95)
**50-68**	HR+	1.71 (0.58;2.93)	3.64 (1.61;5.69)	1.42 (0.37;2.31)	3.21 (1.35;5.29)	9.98 (5.92;12.93)
	HR-	10.42 (7.35;13.62)	4.42 (1.57;6.21)	0.41 (0.01;1.17)	2.71 (1.28;4.02)	17.96 (14.23; 21.03)
	Ratio (+/-)	6.09 (2.08;10.14)	1.21 (0.27;2.19)	0.29 (0.01; 1.21)	0.84 (0.07; 1.69)	1.80 (1.39; 3.01)
**≥69**	HR+	1.71 (0.48;2.89)	4.11 (3.71;5.43)	1.31 (0.26; 2.24)	3.12 (1.18; 5.46)	10.25 (7.07; 13.01)
	HR-	10.82 (7.28;13.59)	3.33 (1.28;5.24)	0.12 (0.01; 1.03)	2.71 (1.29; 4.06)	16.98 (13.24; 20.97)
	Ratio (+/-)	6.33 (1.91; 9.83)	0.81 (0.05;1.51)	0.09 (0.01; 1.09)	0.87 (0.09; 1.78)	1.66 (1.21; 2.85)

Supplementary Table S3.2. Ten year probabilities of death and their corresponding 95% confidence interval according to age and hormone receptor status among patients diagnosed in Stage II.

Age Years		PBC (%, 95%CI)	PCa (%, 95%CI)	PCVD (%, 95%CI)	POC (%, 95%CI)	Total (%, 95%CI)
**≤49**	HR+	9.31 (6.35; 12.32)	2.51 (1.42;5.37)	3.23 (1.21;5.23)	5.14 (3.24;7.15)	20.19 (16.91;24.11)
	HR-	15.12 (12.76;18.67)	2.61 (1.17;4.39)	3.34 (1.05;5.51)	3.32 (1.12;5.36)	24.39 (18.01;30.81)
	Ratio (+/-)	1.62 (2.29; 7.79)	1.04 (0.01;1.95)	1.03 (0.00;1.03)	0.65 (0.25;1.97)	1.21 (1.02;1.81)
**50-68**	HR+	9.01 (6.51;12.91)	2.04 (1.01;3.07)	3.01 (1.35;4.32)	5.01 (3.35;7.21)	19.07 (15.94;22.92)
	HR-	14.11 (10.35;18.52)	2.82 (1.17;3.99)	3.86 (1.01;5.17)	2.43 (1.25;4.15)	23.22 (18.23; 28.10)
	Ratio (+/-)	1.53 (0.89;2.64)	1.21 (0.15;2.08)	1.17 (0.51; 1.79)	0.48 (0.04; 1.49)	1.16 (1.06; 1.83)
**≥69**	HR+	9.61 (6.43;12.39)	2.41 (1.71;3.43)	3.41 (1.22; 5.12)	4.42 (2.11; 6.53)	19.85 (16.07; 21.54)
	HR-	14.65 (10.18;18.99)	2.72 (1.25;3.21)	3.71 (1.58; 5.33)	2.51 (1.39; 4.17)	23.59 (21.91; 26.07)
	Ratio (+/-)	1.52 (1.01; 2.26)	1.13 (0.41;1.81)	1.09 (0.35; 1.79)	0.57 (0.14; 1.76)	1.19 (1.02; 2.74)

Supplementary Table S3.3. Ten year probabilities of death and their corresponding 95% confidence interval according to age and hormone receptor status among patients diagnosed in Stage III.

**Age**<br>**Years**		**PBC**<br>**(%, 95%CI)**	**PCa**<br>**(%, 95%CI)**	**PCVD**<br>**(%, 95%CI)**	**POC**<br>**(%, 95%CI)**	**Total**<br>**(%, 95%CI)**
**≤49**	HR+	32.21 (30.17;34.96)	2.91(0.91;4.86)	3.23 (1.21; 4.27)	3.75 (1.22; 4.78)	42.10 (35.41; 49.12)
	HR-	53.34 (43.19;64.01)	1.23 (0.35;2.31)	3.31 (2.11; 4.14)	3.52 (2.45; 4.51)	61.40 (51.7; 72.91)
	Ratio (+/-)	1.66 (1.05; 1.97)	0.42 (0.03;1.01)	1.02 (0.85;1.35)	0.94 (0.45;1.35)	1.41 (1.12; 1.77)
**50-68**	HR+	32.99 (23.87;42.58)	2.91 (0.87;3.94)	3.11 (1.21; 2.33)	3.82 (2.25;4.88)	42.83 (31.81; 49.03)
	HR-	49.94 (38.77;59.24)	0.94 (0.17; 1.75)	3.31 (1.48;4.72)	2.84 (1.65; 3.47)	57.03 (50.19;60.83)
	Ratio (+/-)	1.51 (1.01;1.99)	0.32 (0.01; 1.09)	1.06 (0.63; 2.25)	0.74 (0.26; 1.23)	1.33 (1.05; 1.71)
**≥69**	HR+	32.91 (22.90;42.93)	3.11 (1.01; 4.97)	3.11 (0.96; 5.08)	4.28 (2.99; 5.73)	43.41 (35.17; 52.12)
	HR-	52.15 (44.32;61.03)	0.54 (0.01; 2.38)	3.25 (0.99; 5.27)	2.77 (1.18; 3.97)	58.71 (49.37;68.74)
	Ratio (+/-)	1.58 (1.03;1.97)	0.17 (0.02; 1.02)	1.05 (0.73; 2.18)	0.65 (0.34; 1.16)	1.38 (0.98; 1.81)

Supplementary Table S3.4. Ten year probabilities of death and their corresponding 95% confidence interval according to age and hormone receptor status among patients diagnosed in Stage IV**.**

**Age Years**	**HR**	**PBC (%, 95%CI)**	**PCa (%, 95%CI)**	**PCVD (%, 95%CI)**	**POC (%, 95%CI)**	**Total (%, 95%CI)**
**≤49**	HR+	70.31 (63.37; 77.93)	12.43 (7.63; 17.29)	3.16 (1.14; 5.19)	3.39 (1.19; 5.17)	89.29 (79.26; 99.18)
	HR-	87.35 (79.72;96.01)	5.57 (1.65; 9.63)	3.33 (1.31; 5.28)	1.44 (0.63; 2.25)	97.69 (87.63; 100.00)
	Ratio (+/-)	1.24 (1.05; 1.57)	0.45 (0.01; 1.96)	1.05 (0.54; 1.85)	0.42 (0.05; 0.98)	1.09 (0.92; 1.98)
**50-68**	HR+	72.61 (62.87; 81.62)	12.22 (5.93; 19.86)	3.43 (1.12; 5.82)	2.76 (1.26; 4.23)	91.02 (81.01; 100.0)
	HR-	86.82 (76.94; 95.99)	5.56 (1.23; 9.28)	3.85 (1.26; 5.93)	1.15 (0.63; 2.17)	97.38 (87.34; 100.0)
	Ratio (+/-)	1.20 (1.01; 1.47)	0.45 (0.01; 1.83)	1.12 (0.73; 1.49)	0.42 (0.15; 1.35)	1.07 (0.65; 1.89)
**≥69**	HR+	70.61 (60.94; 77.63)	13.76 (6.83; 20.06)	2.84 (1.73; 3.92)	4.75 (2.25; 6.96)	91.96 (85.37; 97.26)
	HR-	87.14 (80.19; 95.03)	5.21 (1.27; 8.99)	3.43 (0.91; 5.99)	2.12 (1.01; 3.09)	97.90 (89.99; 100.0)
	Ratio (+/-)	1.23 (1.1; 1.56)	0.38 (0.01; 1.54)	1.21 (0.25; 1.99)	0.45 (0.12; 1.25)	1.10 (0.95; 100.0)

Supplementary Table S4. Causes of death in Catalonia among women during the period 2010-2019.

**Cause of Death**	**(N)**	**(%)**
Infectious and parasitic diseases	93	1.5
Tumours (including BC)	1410	22.2
Diseases of the blood and the hematopoietic organs	40	0.6
Endocrine, nutritional and metabolic diseases	244	3.9
Mental and behavioural disorders	543	8.6
Diseases of the nervous system/organs of the senses	548	8.6
Cardiovascular disease / Circulatory system	1735	27.3
Diseases of the respiratory system	606	9.6
Diseases of the digestive system	308	4.9
Diseases of the skin and subcutaneous tissue	23	0.4
Musculoskeletal system and connective tissue	164	2.6
Diseases of the genitourinary system	239	3.8
Congenital malformations	12	0.2
Symptoms, signs and abnormal findings	160	2.5
External causes of mortality	219	3.4
Total	6344	100.0

N: annual average of the number of deaths; (%): percentage.

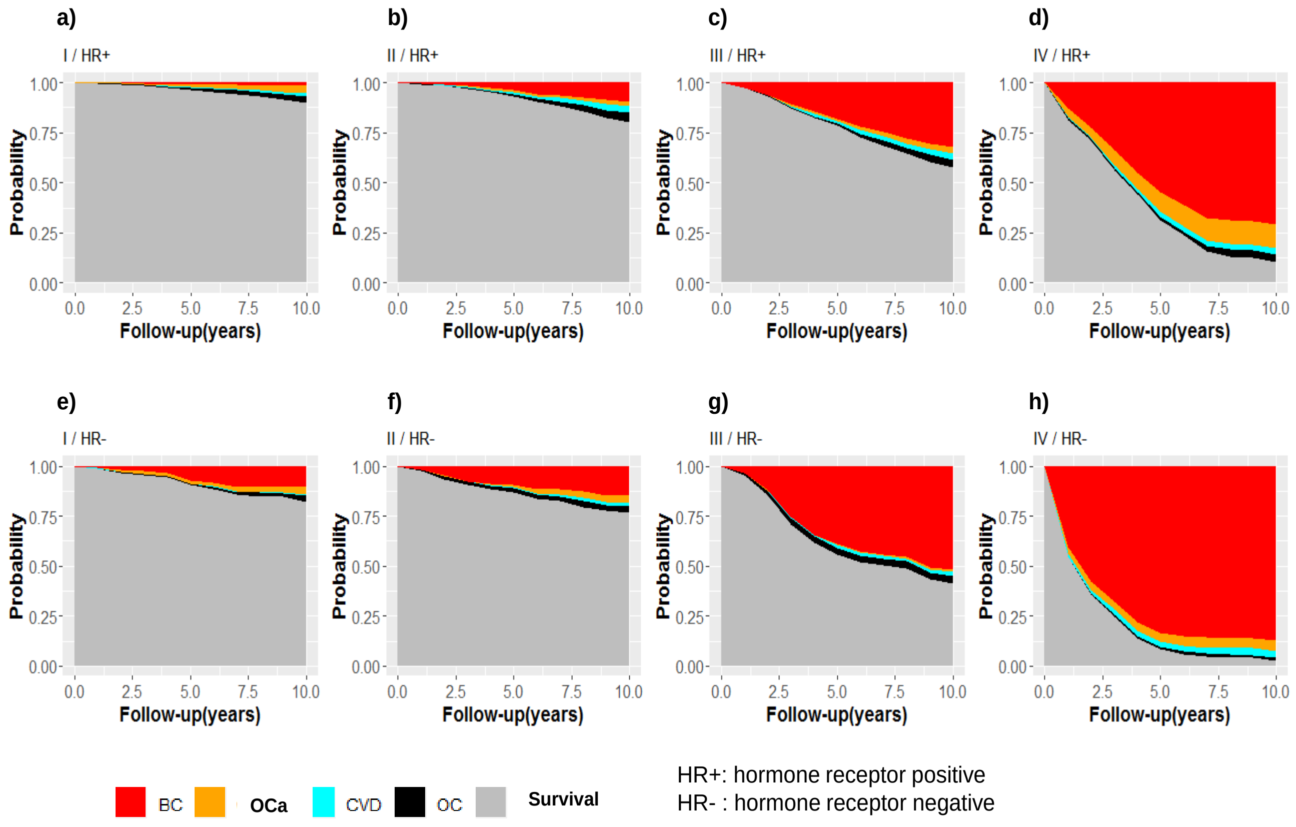

Supplementary Figure S1. Age-adjusted cumulative cause-specific mortality and survival probabilities accross stages of breast cancer at diagnosis and stratifying according to hormone receptor status. (Note: labelling on the panels represent "Stage: from I to IV / Hormone Receptor status: + or -").

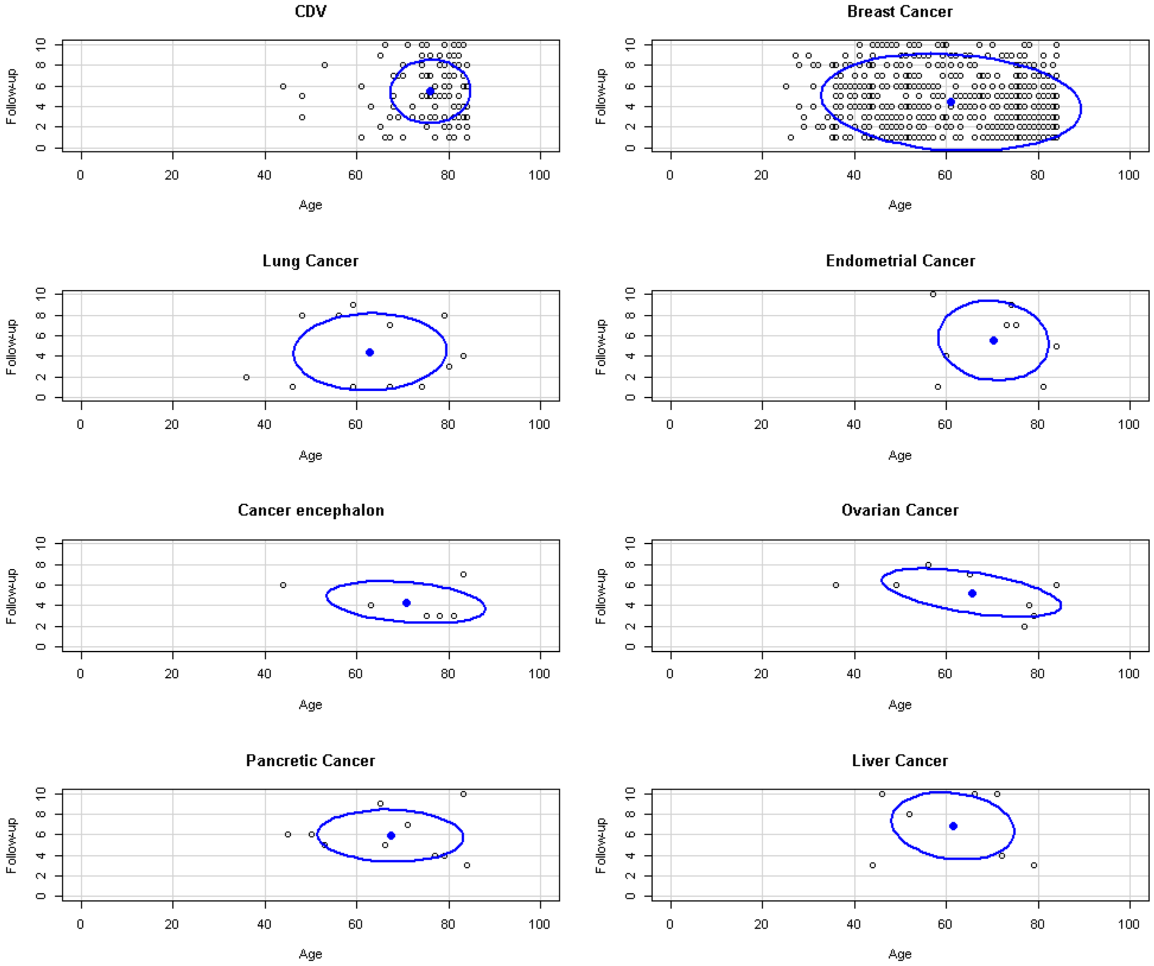

Supplementary Figure S2. Ellipses representing the 95% confidence interval for the age at diagnosis of BC versus the number of years to develop/die from a certain disease (follow-up) for the significant causes of death.

Interpretation: For CVD as the cause of death, the blue point represents the centroid of the 95% joint confidence interval for age and follow-up. This centroid is located beyond 70 years of age (X axis) and 6 years of follow-up. The interpretation is that patients diagnosed of BC who died from CVD were diagnosed beyond 70 years of age (in average) and developed/died from a CVD 6 years after have been diagnosed of BC.
